# Constraints on lunge feeding: krill can clog the baleen of filter-feeding whales

**DOI:** 10.1242/jeb.251852

**Published:** 2026-07-10

**Authors:** Ingrid A. Ackermann, David E. Cade, Jeremy A. Goldbogen, Mark W. Denny

**Affiliations:** Hopkins Marine Station, Oceans Department, Stanford University, Pacific Grove, CA 93950, USA

**Keywords:** Foraging efficiency, Buccal cavity, Pressure, Hydraulic resistance, Parallel resistance

## Abstract

Rorqual whales (Balaenopteridae) grow to exceptional size by lunging to engulf entire swarms of fish or krill, then rapidly concentrating prey using a baleen filter. A 20-m-long fin whale can engulf 60 m^3^ of water and 144 kg of krill in a single gulp and expel the water in 31 s, allowing the animal to make six lunges in a single 8 min dive. But there is a problem hidden in this apparently efficient scheme. Spread uniformly across the 2.9 m^2^ of the baleen window, 144 kg of krill forms a layer 6.3 cm thick. If the accumulated krill clog the filter, it could increase the time needed to expel water, decreasing the rate at which lunges could be made, and negatively impacting foraging efficiency. We devised a model that incorporates measurements of the hydraulic resistance of accumulating krill and the pressure applied to water in the buccal pouch by contraction of muscles and elastic rebound of blubber in the pouch's walls. Given the stress typical of mammalian muscle, the model suggests that approximately 16 min would be required to empty the buccal pouch of an average gulp of krill – twice the duration of typical foraging dives – severely reducing feeding efficiency. Filter time could be reduced to the observed 31 s if a fraction of the baleen window is maintained clear of krill, but it is unclear how that might be achieved. Important questions remain as to how baleen whales feed.

## Teaser

**Highlighted Article:** A model of rorqual whale filter feeding incorporating hydraulic resistance of accumulating krill and buccal cavity pressure suggests it is more complex than a dead-end filter, enabling whales to filter feed on entire swarms of prey.

## INTRODUCTION

Filter feeding has evolved independently multiple times in both vertebrate and invertebrate aquatic animals (Riisgard, 2015; Sanderson and Wassersug, 1993). Various combinations of form and function – often involving arrays of filaments or bristles – have evolved to solve the challenge of filtering food from water (Hamann and Blanke, 2022) at biological scales ranging from microbes to baleen whales (Kirkegaard and Goldstein, 2016; Nielsen et al., 2017; Sanderson and Wassersug, 1993). Taxonomically diverse filter feeders are economically valuable (van der Schatte Olivier et al., 2018), play important ecological roles (Davenport et al., 2000), and their filtration mechanisms drive biomimetic industrial design (Mao et al., 2024; Hamann et al., 2025).

When a filter functions as a sieve, the build-up of captured particles can increase its hydrodynamic resistance, potentially curtailing further filtration. Filter feeders have evolved various morphological and behavioral solutions to avoid this problem. The tube sponge *Aplysina archeri* uses mucus to trap particulate waste that is then shed from their inlet pores using surface contractions, or ‘sneezes’ (Kornder et al., 2022). At high ambient water velocities, barnacles use their filtering structures (cirri) to sieve oncoming flow, but at slower speeds cirri become more paddle-like and actively redirect suspended particles to the mouth rather than acting as a sieve (Trager et al., 1990). Once thought to function as sieves, the gill arches and gill rakers of some filter-feeding fishes instead redirect flow so that suspended particles pass parallel to the filter surface (Brainerd, 2001). Such cross-flow filtration not only decreases the clogging of the filter but also allows the capture of particles smaller than the filter's pore size (Brainerd, 2001; Sanderson et al., 2001). Morphological variation of the filter-feeding apparatus in fish has yielded several variants of cross-flow filtration, including vortical cross-step filtration (Sanderson et al., 2016) and ricochet separation (Divi et al., 2018), both of which involve fluid exiting through pores as particles are retained and concentrated in the main flow. These strategies allow predators to avoid clogging their filters.

Rorqual whales (Balaenopteridae) – such as fin whales (*Balaenoptera physalus*) and blue whales (*Balaenoptera musculus*) – can engulf entire swarms of krill or fish and filter these prey through racks of fringed baleen (Kahane-Rapport et al., 2020). Baleen plates hang down from the roof of the mouth (Fudge et al., 2009; Szewciw et al., 2010), and the fibrous fringes extending from the interior edges of the baleen plates form a porous mat that encircles and walls off part of the mouth opening (Werth et al., 2018). This lunge filter feeding strategy is characterized by several distinct steps (Goldbogen et al., 2017): (1) acceleration of the whale towards a prey aggregation, (2) mouth opening and the engulfment of water and suspended prey, (3) nearly complete mouth closure around the engulfed water and prey such that the jaws are slightly apart – leaving a baleen ‘window’ (the internal filter surface area) as the interface between fluid inside and outside the mouth, (4) the purging of the mouth as the water passes through the baleen ‘window’ and the consequent filtering of prey that is kept inside the mouth, and (5) deglutition.

Although cross-flow filtration and other mechanisms have been hypothesized for rorqual whales (Goldbogen et al., 2007), the exact processes remain poorly understood (Werth and Potvin, 2024). Regardless of mechanism, it seems likely that sieved prey will eventually accumulate on the baleen surface as water exits the mouth (Werth, 2001). Therefore, the potential clogging from prey accumulated on the filter could increase resistance to flow. Increased resistance would in turn slow the filtering process or increase the mechanical energy needed from muscles to empty the buccal pouch. Both factors could negatively impact the energetic efficiency of foraging. However, the resistance to flow from accumulated prey has never been investigated in baleen whales.

Here, we combined data from biologging, numerical models and experimental measurements to explore the extent to which prey may cause clogging during rorqual's intermittent filtration process. We ask two questions. First, what pressure can the contraction of muscles and the elastic rebound of blubber apply to water in the buccal cavity? Second, is that pressure sufficient to expeditiously expel water against the hydraulic resistance of krill stacked against the baleen filter? Our results suggest that filter clogging could potentially limit the efficacy of lunge feeding, and we explore mechanisms by which whales have avoided those constraints to maximize efficiency.

## METHODS

### Estimating buccal pressure

After a feeding gulp, water is squeezed from the buccal cavity as contraction of muscles in the pouch wall and the elastic recoil of the ventral groove blubber (VGB) pressurize the cavity relative to the surrounding water. To estimate the pressure that can be applied to expel water, we made several simplifying assumptions, as detailed below.

#### Pouch morphology

**Fig. 1.**
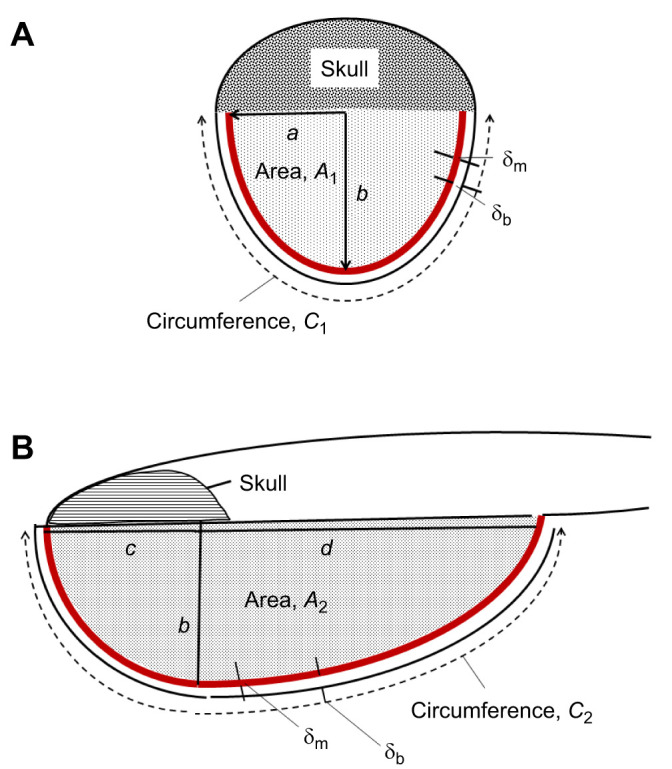
**A schematic representation of the buccal pouch at full extension after a feeding gulp.** (A) A transverse section through the skull and buccal pouch of a rorqual whale. The area enclosed by the pouch is approximately half that of an ellipse with semi-axis *a* (equal to half the skull width) and semi-axis *b* (equal to the length of the lower jaws in lateral projection, or *L*_jaw_). Semi-axis *c* (also equal to *L*_jaw_) is not shown; it comes out of the page toward the reader. The thickness of the oblique muscle (δ_m_) and of the ventral groove blubber (δ_b_) is shown (not drawn to scale). (B) A mid-longitudinal (sagittal) section; *c* is equal to *L*_jaw_; *d* is the distance from the maximal extension of the pouch to its posterior end near the umbilicus.

Because they represent one of the best studied rorqual species from both an anatomical and a functional perspective, fin whales provide a useful model species for our calculations. We describe the morphology of their buccal pouch following the model of Shadwick et al. (2013) (Fig. 1). Here, the anterior half of the pouch is assumed to be a quarter ellipsoid with its transverse semi-axis (*a*) equal to one-half of the bizygomatic width, or the width of the head at the temporomandibular joint (1.09 m; Fig. 1A) and its longitudinal semi-axis (*c*) equal to the length of the jaws (*L*_jaw_; as in Potvin et al., 2009; Goldbogen et al., 2009), or specifically the laterally projected length of the lower mandibles (3.98 m; Fig. 1B) (see Table 1). The ellipsoid's third semi-axis (*b*) varies in length. When the pouch is at rest, its length is equal to the width of the head; when the pouch is inflated after a gulp, *b* has increased to the length of *L*_jaw_ (as shown in Fig. 1A,B). The posterior half of the pouch is modeled as a quarter ellipsoid with its transverse semi-axis (*a*) equal to head width, its longitudinal semi-axis (*d*) equal to the distance from the temporomandibular joint to the umbilicus (7.22 m; Fig. 1B), and a third semi-axis (*b*) that again varies from head width with the pouch not inflated to jaw length after the pouch inflates (Fig. 1B). The estimated surface area of the pouch (the combined surface area of the two quarter ellipsoids) varies from 30.5 m^2^ when at rest to 79.2 m^2^ when inflated, an increase of 2.6-fold.

**Table 1.** Definition and value of symbols and abbreviations

Symbol	Units	Value	Definition
*A*	m	1.09	Half head width (Shadwick et al., 2013)
*A*_1_	m^2^	6.81	Transverse area
*A*_2_	m^2^	35.0	Longitudinal area
*b*	m	3.98	Length of lower jaws in lateral projection (drawn 90 deg open from the closed position), *L*_jaw_ (as in Shadwick et al., 2013)
*c*	m	3.98	Length of lower jaws in lateral projection (mouth closed), *L*_jaw_ (as in Shadwick et al., 2013)
*C*_1_	m	9.16	Transverse circumference
*C*_2_	m	15.5	Longitudinal circumference
*d*	m	7.22	Longitudinal ellipsoid semi-axis
***g***	m s^−2^	9.81	Gravity
*H*	m		Hydraulic head
*m*_k_	kg	90, 40−180	Mass of krill
*P*	Pa		Pressure in pouch
*R*	Pa s m^−1^		Resistivity
α		2.4	Exponent
β	degrees		Muscle angle
δ_b_	m	0.046	Blubber thickness (Shadwick et al., 2013)
δ_m_	m	0.046	Muscle thickness (Shadwick et al., 2013)
δ_k_	m		Krill thickness
ρ	kg m^−3^	1000	Water density
ρ_k_	kg m^−3^		Krill mass/gulp volume
ρ_p_	kg m^−3^	784	Krill packing density
σ	Pa		Stress

#### Blubber and muscle thickness

The VGB has a thickness, δ_b_, of approximately 12 cm at rest (Shadwick et al., 2013). Assuming that the blubber's volume does not change when the material is deformed (Orton and Brodie, 1987), its thickness is reduced 2.6-fold to 4.6 cm when the pouch is maximally inflated. Similarly, the average thickness of the oblique stratum (OS) of muscle is 12 cm at rest (Shadwick et al., 2013), which (assuming the muscle is isovolumetric) decreases to δ_m_=4.6 cm when the pouch is inflated. (Although the OS muscle tapers along the buccal cavity from the lower mandible to the end of the VGB, we use its average thickness in the extended pouch.) The pouch is also lined by a stratum of longitudinal muscle (LS) that extends along the anterior half of the pouch's length, but we assume that LS does not contribute to the pressure needed to empty the pouch. Orton and Brodie (1987) suggest that, given its morphology and the fact that it consists solely of slow twitch fibers, LS's only function is to play a postural role in pouch morphology when the pouch is empty.

#### Contractile forces

When stretched, the VGB can apply force in both the longitudinal and circumferential directions. Shadwick et al. (2013) estimate that when the buccal pouch is inflated during a gulp, the VGB reaches an extension ratio (stretched length/resting length) of 1.38 in the longitudinal direction and 2.58 in the circumferential direction. At these extensions, information presented by Orton and Brodie (1987) (see their fig. 5) suggests that the stresses (force per cross-sectional area) exerted by the VGB are σ_L,b_=15.9 kPa in the longitudinal direction and σ_C,b_=53.6 kPa in the circumferential direction.

By contracting, the OS muscles can apply force to the water in the pouch. Because the fibers in this layer lie in a cross-striated pattern at an angle ±β to the longitudinal axis of the pouch (Orton and Brodie, 1987), when contracting they apply force in both the longitudinal and circumferential directions (see Appendix 1). β depends on the relative forces applied by muscle and blubber (see Appendix 2).

The combined pressure imposed by muscles and blubber in the inflated pouch can be estimated from two perspectives (Fig. 1). Fig. 1A shows a schematized transverse section through the skull and inflated buccal pouch at the location of the pouch's maximum breadth. In our model of pouch morphology, the area of water enclosed within this cross section, *A*_1_, is equal to half the area of an ellipse (π*ab*/2); *A*_1_=6.81 m^2^. The circumference of the pouch in this section, *C*_1_, is half the circumference of the ellipse 
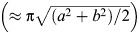
; *C*_1_=9.16 m.

Blubber with a transverse cross-sectional area δ_b_*C*_1_ exerts a longitudinal force:
(1)

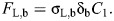


Muscle with a transverse cross-sectional area of δ_m_*C*_1_ applies a longitudinal force (Appendix 1):
(2)

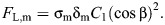


Here, σ_m_ is the stress (force per area) maintained by the contracting muscle. The pressure resulting from combined elastic and muscular force is:
(3)

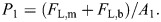


Fig. 1B shows a schematized mid-longitudinal (sagittal) section through the skull, body and inflated buccal cavity. In this perspective, the area enclosed within the cross section, *A*_2_, is the sum of a quarter-circular anterior area (π*cb*/4= π*c*^2^/4), plus a quarter-elliptical posterior area (π*bd*/4); *A*_2_=35.0 m^2^. Pressure is applied to this area by both circumferential recoil of the VGB and contraction of the muscles.

Circumferential force applied by the recoil of the VGB is:
(4)

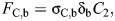
where *C*_2_ is the circumference of the section; *C*_2_ is 15.5 m. Circumferential force applied by contracting oblique muscles is (Appendix 1):
(5)

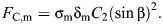


The combined circumferential contribution to pressure from muscle and blubber is:
(6)

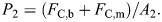


According to basic fluid statics, because *P*_1_ and *P*_2_ are applied to the same water, they must be equal, which allows us to solve for the angle β at which muscle fibers must act (Appendix 2). To our knowledge, the force per area exerted by contracting OS muscle has not been measured. Consequently, we used a range of values from 200 kPa (the average mammalian value cited by Rospars and Meyer-Vernet, 2016) to 1000 kPa, the maximum value cited by Vogel (2001). The calculations from our model suggest that the buccal pouch can be squeezed to a pressure of 4.1 kPa by blubber and average muscle contraction, and a pressure of 17.7 kPa by blubber and maximal muscle contraction. For average contraction stress, muscle fibers are oriented with β=58.7 deg; for maximum contraction stress, β=59.8 deg.

In theory, it would be possible to estimate buccal pressure from a third perspective involving a coronal plane perpendicular to the transverse and sagittal planes used here. However, productive use of that plane would require additional anatomical details that are not currently available (see Appendix 3).

### Hydraulic resistance

To determine how prey (i.e. krill) clogging could affect the dynamics of filtration, we designed an experiment to simulate the resistance krill impose to flow as they accumulate on baleen during the filtration stage of lunge feeding. To that end, we first estimated the thickness of krill that would accumulate and then measured the hydraulic resistance that thickness would create.

#### Krill thickness

A 20-m-long fin whale typically engulfs 60 m^3^ of water in a feeding gulp, and in 31 s this volume is expelled through a baleen ‘window’ of 2.9 m^2^ (Werth et al., 2018). The resulting average exit velocity of the purged water during the filter phase is 0.67 m s^−1^ (Kahane-Rapport and Goldbogen, 2018; Werth et al., 2018). The mass of krill in the engulfed volume depends on prey density and the whale's feeding behavior, both of which have been estimated and calculated in previous work. In particularly dense patches, blue whales (similar in morphology and dive behavior to fin whales) fed on krill with an average density of 1.49 kg per cubic meter of seawater (with a geometric standard deviation of 1.6) (Cade et al., 2021). Thus, assuming a mean body size-specific engulfment capacity (60 m^3^), we calculated a mean engulfed prey mass (*m*_k_) in ideal feeding conditions of 90 kg with a range of mean experienced values from 40 to 144 kg. We obtained frozen Antarctic krill from a pet shop (*Euphausia superba*, San Francisco Bay Brand); the krill were well preserved and other than being inanimate were lifelike. We found that 100 ml of krill (loosely packed as they would be on the baleen) had a mass of 78.4 g, and therefore a packing density, ρ_p_, of 784 kg m^−3^. Dividing engulfed mass by packing density, we estimate that an average gulp has a krill volume of 0.115 m^3^ with a calculated range (±s.d.) from 0.072 to 0.184 m^3^. Spread evenly across the baleen ‘window’ area (2.9 m^2^), this suggests that krill could accumulate to an average thickness of 4.0 cm (2.5 to 6.3 cm).

#### Krill hydraulic resistance

**Fig. 2.**
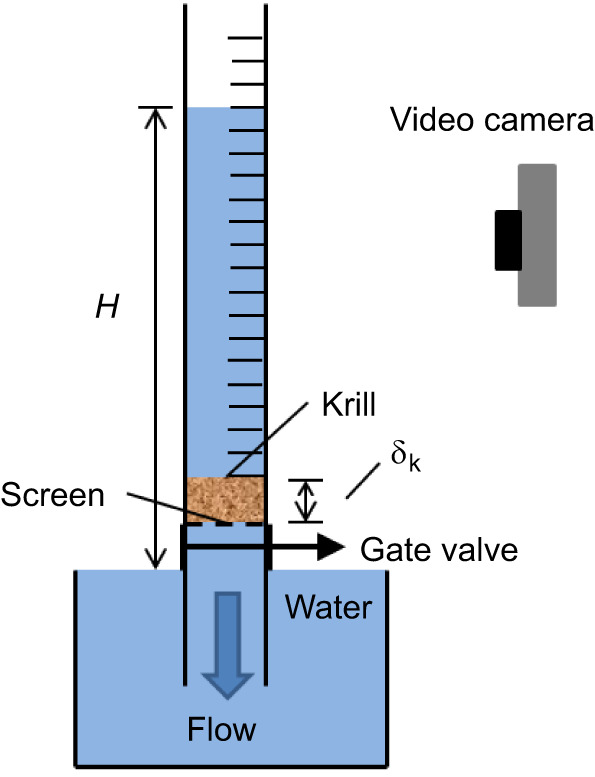
**A schematic representation of the apparatus used to measure krill's resistance to flow.** *H* is the reference height of the water column's meniscus, which decreases as water flows out; δ_k_ is the thickness of the layer of krill.

To measure the resistance that sieved krill impose on the outflow of water, we constructed the gravity-powered apparatus shown in Fig. 2. A vertical tube made from clear acrylic plastic (1.6 m tall, 0.1 m inside diameter) was attached to a gate valve at its lower end, and a circle of woven steel mesh was placed above the valve to simulate the fibrous mat of the whale's baleen [10 pores per inch (25.4 mm), wire diameter 0.75 mm]. With the valve closed, the lower end of the tube was positioned in a full, open-topped tank of freshwater (density, ρ, 1000 kg m^−3^). Freshwater was then added to fill the tube. When the gate valve was rapidly opened, hydrostatic pressure (ρ***g****H*) forced water to flow out of the tube and into the tank, where it overflowed the sides. Here, ***g*** is acceleration due to gravity (9.81 m s^−1^) and *H* is the instantaneous height of the column's meniscus above the tank's water level. By video recording the fall of the water column relative to fiducial marks on the tube (240 frames s^−1^), we could track the speed (m s^−1^) at which water flowed through the mesh as a function of hydrostatic pressure. During these video measurements, pressure ranged from 8.9 to 12.4 kPa, within the range of buccal cavity pressures estimated from our model. The experiment was then repeated with a layer of krill above the mesh. Three thicknesses of krill were tested – 1.9, 3.7 and 5.6 cm – nearly spanning the expected range in fin whales.

## RESULTS: PREDICTED FLOW RATES

**Fig. 3.**
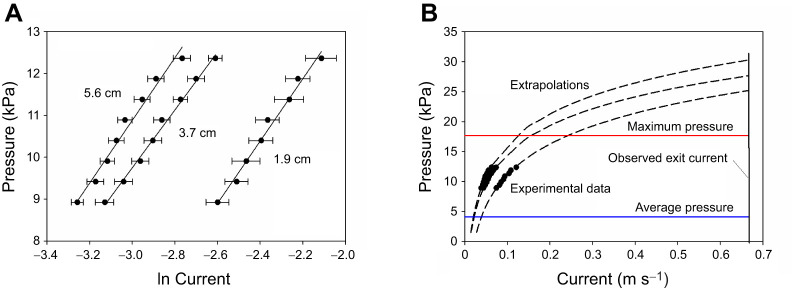
**The pressure required to force water through a layer of krill increases with the speed of the outward current, *u* (m** **s^−1^).** (A) For a layer of krill 1.9 cm thick, pressure (in kPa) *P*=7.43×ln*u*+28.22 (*r^2^*=0.987). For a layer 3.7 cm thick, *P*=6.90×ln*u*+30.45 (*r^2^*=0.994). For a layer 5.6 cm thick, *P*=7.48×ln*u*+33.32 (*r^2^*=0.977). Data are means±s.e.m. For krill layer thicknesses of 1.9, 3.7 and 5.6 cm, we replicated the experiment 4, 6 and 5 times, respectively. (B) The data can be extrapolated to estimate current at average and maximal buccal pressure and the pressure required to achieve the observed current of 0.67 m s^−1^.

Pressure required to force water through a layer of krill (corrected for the negligible resistivity of the bare screen, 1.9×10^−4^ Pa s m^−1^) increases approximately linearly with the natural logarithm of the speed of outward flow (Fig. 3A; *r*^2^>0.97 in each case). Extrapolating these trends allows us to predict speed at the pressure applied by blubber and average muscle contraction (Fig. 3B): 0.039 m s^−1^ for a layer 1.9 cm thick, 0.022 m s^−1^ for a layer 3.7 cm thick and 0.020 m s^−1^ for a layer 5.6 cm thick. All three predicted speeds are far less than the 0.67 m s^−1^ required to empty the pouch in the observed 31 s. When muscle contraction is maximal, predicted exit speeds are: 0.243 m s^−1^ for a layer 1.9 cm thick, 0.157 m s^−1^ for a layer 3.7 cm thick and 0.124 m s^−1^ for a layer 5.9 cm thick; again, far below the observed exit current of 0.67 m s^−1^.

These currents can be used to estimate the time required to expel water from the buccal pouch. For example, for average pressure and a 3.7 cm-thick layer of krill, volumetric flow rate through the baleen window is equal to the product of outward current (0.022 m s^−1^) and the area of the baleen filter (2.9 m^2^): 0.064 m^3^ s^−1^. At this rate, it would take 940 s (15.7 min) to empty 60 m^3^ from the pouch, nearly twice the duration of a typical foraging dive. For maximal pressure, outward flow rate is 0.157 m s^−1^, volumetric flow rate is 0.455 m^3^ s^−1^, and it would take 132 s (2.20 min) to empty the pouch.

Our experimental data can be extrapolated farther to estimate the pressures that would be required to achieve the average outward current in a fin whale (0.67 m s^−1^; Fig. 3B): 25.2±1.7 kPa (±95% confidence limit, CL) at a thickness of 1.9 cm, 27.7±1.3 kPa at a thickness of 3.7 cm and 30.3±3.0 kPa at a thickness of 5.6 cm (Fig. 3B). These pressures are nearly twice that supplied by elastic rebound of the VGB in combination with our assumed maximal muscle contraction stress.

Muscles could potentially contract with greater force than we estimate. However, in combination with the elastic rebound of the blubber, the muscle stresses required to create the pressures needed to empty the pouch in 31 s are 1305, 1466 and 1632 kPa for layers 1.9, 3.7 and 5.6 cm thick, respectively (see Appendix 4). All these stresses are substantially greater than that of our estimated maximal contraction (1000 kPa; Vogel, 2001).

Our calculations could be in error owing to our simplifying assumption which posits, unrealistically, that all krill have accumulated on the baleen before any water is expelled. Barring cross-flow or tongue-clearing effects (discussed below), krill are likely to be distributed evenly throughout the water inside the buccal pouch, and will accumulate gradually as volume is expelled. The low initial thickness of krill could allow water to be expelled against less resistance than we have calculated; therefore, a lower pressure would be required to empty the buccal cavity in the time allowed. However, this effect is unlikely to be sufficient to reconcile our measurements of hydraulic resistance with empirical observations.

**Fig. 4.**
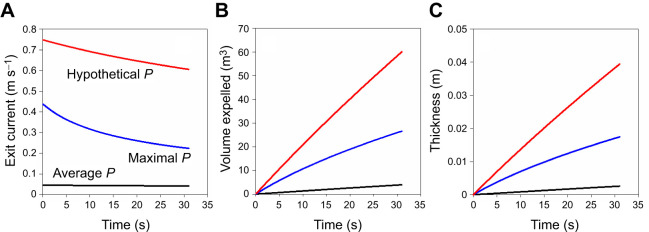
**The effects of gradual krill accumulation on buccal pressure (*P*) with average muscle contraction (black lines), maximal muscle contraction (blue lines) and the hypothetical pressure required to empty the pouch in 31 s (red lines).** (A) Flow velocity decreases rapidly as krill accumulate. (B) The volume expelled by average and maximal pressure is less than the volume engulfed (60 m^3^). (C) The thickness of the krill layer increases through time.

**Fig. A3.**
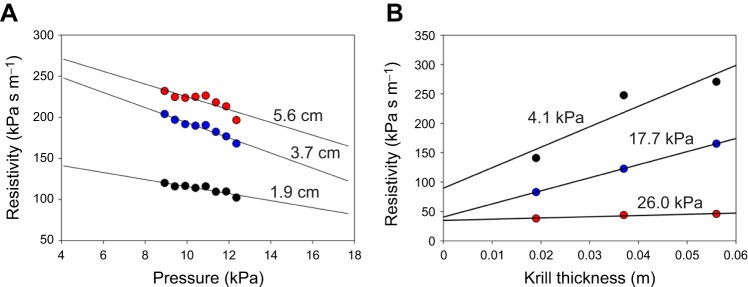
**Hydraulic resistivity of the krill as a function of pressure and krill thickness.** (A) Measured hydraulic resistivity for the three experimental thicknesses of krill (1.9, 3.7, 5.6 cm), each as a function of pressure. The solid lines are least-squares fits to the data, allowing us to estimate resistivity (*R* in kPa s m^−1^) at the pressures (*P* in kPa) applied by the buccal pouch wall (4.1 and 17.7 kPa). For a krill layer 5.6 cm thick, *R*=−7.75×*P*+302.4 (*r*^2^=0.726). For a krill layer 3.7 cm thick, *R*=−9.20×*P*+285.3 (*r*^2^=0.942). For a krill layer 1.9 cm thick, *R*=−4.27×*P*+158.4 (*r*^2^=0.835). (B) Resistivity (*R* in kPa s m^−1^) as a function krill thickness (δ_k_ in m) at the pressures applied by the buccal pouch and the hypothetical high pressure required to empty the pouch in 31 s. Lines are linear fits to the data. For a pressure of 4.1 kPa, *R*=229.0×δ_k_+89.61 (*r*^2^=0.876). For a pressure of 17.7 kPa, *R*=129.4×δ_k_+40.46 (*r*^2^>0.999). For a pressure of 26.0 kPa, *R*=43.1×δ_k_+34.77 (*r*^2^>0.918).

In Appendix 5, we calculate how the increasing thickness of a uniformly spread krill layer interacts with the outward flow of water for the pressures applied by the buccal pouch wall, with the results shown in Fig. 4. Flow is initially rapid, but quickly declines (Fig. 4A) because our measurements suggest that even a thin layer of krill can create a substantial resistance to flow (see Appendix Fig. A3B). As a result, time-averaged flow rates for the pressures that the buccal-pouch wall can provide (both average and maximal) are far below the 0.67 m s^−1^ needed to empty the pouch in the time observed (0.04 and 0.30 m s^−1^, respectively). The volume expelled in 31 s by average and maximal pressure (3.9 and 27 m^3^, respectively) is substantially less than the 60 m^3^ engulfed (Fig. 4B), and the corresponding accumulation of krill is likewise less (Fig. 4C). To empty the pouch in 31 s against a gradually accumulating layer of krill would require a pressure of 26.0 kPa. Perhaps surprisingly, this is only slightly less than that needed if the krill accumulated instantaneously (27.7 kPa). The small difference is again due to the fact that, apparently, even a thin layer of krill offers substantial resistance (Fig. A3B). Future measurements of the resistance offered by thin layers of krill and the bare baleen itself (including any flow-dependent variation in the resistance of the flexible baleen or live krill) will help to refine estimation of the effects of gradual accumulation of krill.

## DISCUSSION

Filter feeding allows animals to process vast amounts of prey-laden water, thereby achieving high-energy intake rates at low trophic levels. To evaluate the efficacy (e.g. the energetic efficiency) of filter feeding, these intake rates must be compared with the costs of filtration. For a passive filter feeder such as a barnacle, the cost of filtration is low, and the animal can survive in a dilute soup (i.e. low prey density). By contrast, the energetic cost of lunge feeding by rorqual whales – the taxa of focus in this study – is high because the animals' extraordinary mass must be accelerated from rest in each feeding event (Goldbogen et al., 2007, 2008). The high suspected cost of feeding has led researchers to hypothesize that minimum prey density thresholds may be required to support the high metabolic demands at this extreme organismal scale (Goldbogen et al., 2011). However, after a short-duration high-energy lunge, the extended filtering time that follows is characterized by limited locomotor activity (Kahane-Rapport et al., 2020). Consequently, metabolic rates during foraging based on respiratory rates are quite low (Videsen et al., 2023), and at the largest body sizes the energy use during foraging is only slightly higher than that during non-foraging periods (Blawas et al., 2025). Therefore, rorqual whales appear to exhibit a high-cost feeding mechanism while simultaneously achieving low metabolic rates during foraging.

It has been suggested (Goldbogen et al., 2011; Sims, 1999) that because lunge feeding maximizes prey density in the engulfed volume, it more than compensates for the cost of acceleration, allowing rorquals to achieve exceptional size. However, these analyses implicitly assume that high-density aggregations of food particles are filtered with the same effort as low-density aggregations. If high-density food increases the resistance to filtration because of clogging (as our data suggest), it would require greater energetic effort to filter the engulfed volume in a given time or necessitate a longer time to filter the volume, thereby increasing the interval between lunge feeding events. In either case, the advantages of lunge feeding could be compromised. Our experiments suggest that krill accumulating on baleen can substantially increase the hydraulic resistance encountered while processing an engulfed volume of prey. Indeed, our calculations suggest that the combined efforts of muscles and blubber are not sufficient to empty the buccal pouch in the interval observed in nature. For an average gulp of krill, the interval would be increased by a factor of approximately 30 given average muscle stress (to twice the duration of a typical dive), and even for maximal muscle stress, the interval would be increased by a factor of approximately 4. How can we reconcile our measurements with empirical observations? There are several possibilities, each of which provides guidance for future research, as detailed below.

### Parallel resistance

**Fig. 5.**
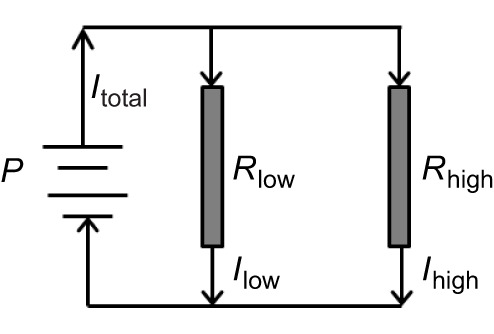
**By analogy to an electrical circuit, flow through parallel resistances in a whale's buccal cavity is governed by the relative resistivity of nearly bare (*R*_low_) and clogged (*R***_high_**) baleen.** *P*, pressure (analogous to voltage); *I*, current.

Our calculations for the pressure required to expel water through a layer of krill assume that krill are evenly spread across the baleen area. That need not be so, in which case our calculations need to be adjusted. To explore that possibility, we assume that a fraction of the baleen area has a sparse accumulation of krill, offering a lower resistance to flow while the remaining krill accumulate to a greater thickness on the remaining area. To calculate the pressure required to expel water at volumetric flow rate *I*_total_=d*V/*d*t* through a baleen window with area *A*_total_, we resort to an analogy to an electrical circuit (Fig. 5). Pressure *P* (analogous to voltage) drives flow through parallel arms of the circuit with the resistivity (resistance per filter area) of the nearly bare baleen *R*_low_ (associated with area *A*_low_) and the thickly accumulated krill *R*_high_ (associated with area *A*_high_=*A*_total_−*A*_low_). Flow in each arm of the circuit is set by that arm's resistance: *I*_low_=*PA*_low_/*R*_low_, *I*_high_=*PA*_high_/*R*_high_. Overall flow is the sum of the flows in the two arms:
(colorred7)

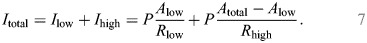
Solving for *P:*
(colorred8)

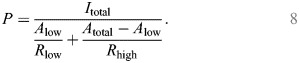


**Fig. 6.**
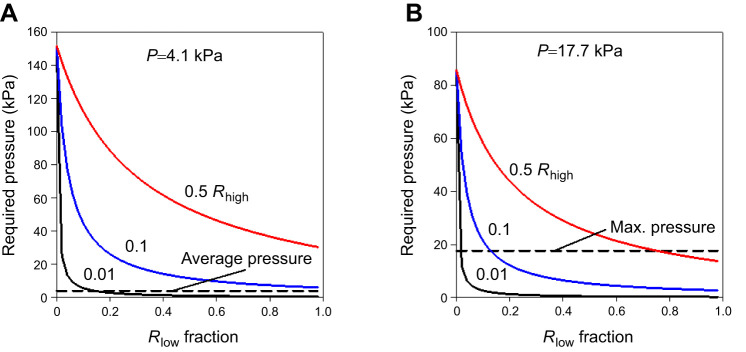
**The effect of parallel resistance on the pressure required to expel water through an average thickness of accumulated krill.** (A) The effect using the resistivity measured when average pressure (4.1 kPa) is applied to water in the buccal pouch (Fig. A3B). Colored lines represent assumptions of the resistivity of the lower-resistivity baleen area where water would exit, where *R*_low_ is 1% (black), 10% (blue) and 50% (red) that of a 4-cm-thick layer of krill. (B) The effects using the resistivity measured when maximal pressure (17.7 kPa) is applied.

The pressure required to empty the pouch in a given interval is sensitive to *R*_low_, the resistivity of the nearly bare baleen. If *R*_low_ is small relative to *R*_high_, even a minor fraction of bare baleen can substantially reduce the pressure needed to expel a gulp in 31 s. Fig. 6 illustrates this effect. Given the resistivity estimated for average pressure (Fig. A3B), Fig. 6A shows the pressure that would need to be applied to water in the buccal pouch as a function of the fraction of the baleen window that is in a low-resistance state. When *R*_low_ is one-hundredth that of a thick layer, approximately 15% of the baleen must be maintained in that low-resistance state for full expulsion in 31 s. If *R*_low_ is half or even one-tenth that of a thick layer, average pressure is insufficient to efficiently empty the pouch. The situation is only slightly improved assuming the resistivity estimated for maximal pressure applied to water in the buccal pouch (Fig. 6B). If *R*_low_ is half that of a 3.7 cm-thick layer, the pouch can be emptied in 31 s, but only if approximately 75% of the baleen window is in the low-resistance state. If *R*_low_ is one-tenth that of a thick layer, more than 10% of the baleen must still be maintained in the low-resistance state.

But how small is *R*_low_ in a whale? We have not directly measured the resistivity of very thin krill layers. It seems reasonable, however, that the thin layer of even a sparse accumulation will result in high resistivity. By analogy, imagine a filter consisting of *x* circular holes in an otherwise solid membrane, and the effect of spherical food particles on the resistivity of that filter. If the diameter of the spheres is greater than that of the holes (such that the membrane acts as a sieve), filtering as few as *x* particles would fill every hole, completely clogging the filter. Similarly, a few krill, each of which is substantially larger than the spacing between bristles in the baleen window, could effectively increase the resistivity of even sparsely covered baleen. Linear fits to our measured resistivities (Fig. A3B) suggest that even very thin layers of krill would have a resistivity 30–81% that of a 3.7 cm-thick layer. Note that our model assumes that all krill accumulate on the baleen window. If, instead, krill accumulate uniformly on the entire inner surface area of the contracted pouch and baleen (30.5 m^2^), they would form a layer 0.38 cm thick, and would still have an estimated resistivity greater than 30% that used in our calculations.

In summary, the effects of parallel resistance provide a potential means by which whales can reduce the pressure needed to effectively filter krill. However, that potential depends on their ability to maintain a substantial fraction of the baleen window clear (or nearly clear) of accumulating prey.

Goldbogen et al. (2007) hypothesize that flows within the buccal pouch could act to set up a form of cross-flow filtration in which food particles move tangential to the baleen while water moves normal to the filter. If such flows exist, they could provide a mechanism for keeping some of the baleen window clear of prey, thereby lowering the parallel resistance of the filtering apparatus. Diver footage of foraging blue whales shows that the engulfed water mass may be actively or passively moved longitudinally within the oropharyngeal cavity (fig. 8 in Goldbogen et al., 2017; see https://drive.google.com/file/d/10Ls6_mFf3TsU6-01Xb2zW5LxpgnAptN3/view?usp=sharing, courtesy of Hugh Pearson, Silverback Films, BBC film ‘The Hunt’ 2015, NMFS Permit #16111). Such motion may serve to keep suspended prey from building up along the baleen surface and thus avoid high-resistance clogging.

Similarly, our model does not address the role(s) that the tongue might play in feeding. This massive and highly elastic organ could potentially be used to keep krill from accumulating on the baleen window during expulsion (a necessity suggested by our model) and to consolidate the engulfed prey so they can be swallowed. But as the whale engulfs a volume of water, the tongue inverts backwards towards the umbilicus. It is not readily apparent how the tongue might subsequently clear krill from the baleen; this remains a question for further research.

Future measurements of the resistivity of nearly bare baleen, modeling of flows within a contracting buccal pouch, and assessment of the tongue's role will be required to address the potential for parallel resistance to explain how rorqual whales are able to filter high numbers of small-bodied prey such as krill (Hamner and Hamner, 2000) from extraordinarily large engulfment volumes.

### Pouch contraction

In our calculations, we examined the interaction among pressure, flow and krill-induced hydraulic resistance when the buccal pouch is maximally filled. A more exact model of pouch emptying would include the effects of blubber and muscle contraction as the pouch empties. As the pouch wall contracts, its thickness increases (such that, for a given stress, it can exert more force) and the cross-sectional area of water to which its force is applied decreases (increasing the pressure exerted by a given force). This interaction potentially increases a whale's ability to expel water.

However, the interaction between these two factors is sensitive to how the stresses in blubber and muscle vary with their extension. The force–extension curve for buccal muscle is (to our knowledge) unknown, so it would be difficult to accurately model the muscle's contribution as the pouch contracts. However, in general, once muscles have contracted by a fraction of their stretched length, their force decreases as length decreases further, even though cross-sectional area increases (Vogel, 2001). Additionally, force decreases as the speed of contraction increases (Vogel, 2001). Both these properties suggest that as buccal muscles contract during expulsion, the pressure they impose should be less than that estimated in our models. Furthermore, a simple heuristic model suggests that pressure applied by blubber will similarly decrease as volume reduces (water exits), making it even less likely that the pouch can be emptied in the time required.

In Appendix 6, we use the measured mechanical properties of blubber to estimate how pressure *P* in a cylindrical vessel (of resting radius *r*_0_) varies as a function of expanded radius *r*:
(colorred9)

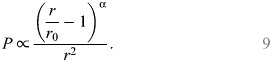


**Fig. 7.**
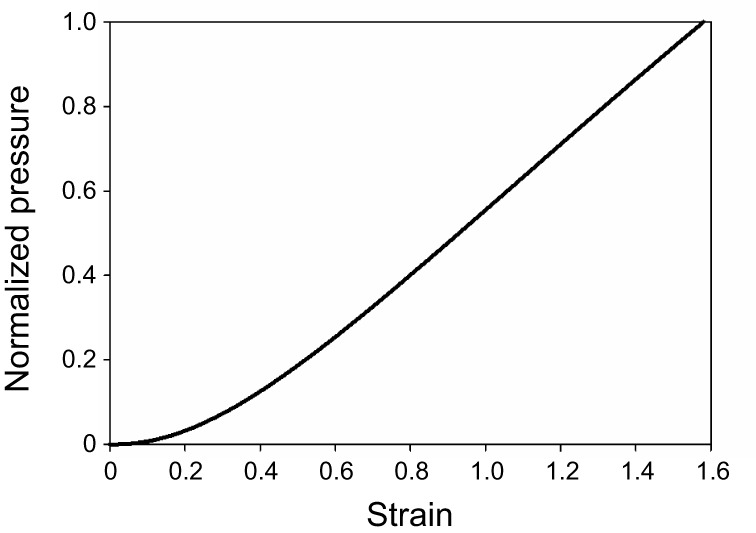
Normalized pressure in a closed cylindrical vessel made from blubber as a function of the strain of the vessel's wall material.

For fin-whale VGB stretched circumferentially to strains <2, α is approximately 2.4 (see fig. 5 of Orton and Brodie, 1987). [Note that Orton and Brodie (1987) give their stress–extension curves in terms of true strain. True strain is the natural logarithm of extension ratio.]. Setting α=2.4, pressure (normalized to the pressure at maximum extension ratio in the buccal pouch) is shown in Fig. 7 as a function of strain. Pressure exerted by the blubber increases as the pouch fills and the blubber stretches.

In a whale, this process runs in reverse during filtration. The pouch is stretched to some extension ratio during a gulp (strain ∼1.6). The pressure exerted by the blubber then decreases as the pouch deflates, making it even more difficult to force water through the accumulating krill. This conclusion is amplified by the observation that in dead fin whales the buccal pouch is flaccid, indicating that elasticity alone is insufficient to restore the pouch to its contracted, streamlined shape (R. E. Shadwick, personal communication).

### Longitudinal muscle

In our calculations, we followed the suggestion of Orton and Brodie (1987) that the longitudinal muscles in the pouch do not contribute to the expulsion of water. If, instead, contraction of the LS contributes to intra-pouch pressure during filtration, it could help to alleviate the impact of krill clogging. The potential magnitude of that contribution is difficult to estimate, however. The longitudinal muscles extend only along the anterior half of the buccal pouch. To apply pressure to water in the pouch, the LS must act in series with the posterior material through which it is connected to the body, and pressure will depend on the compliance of that material. Only if the connecting material is very stiff can a substantial pressure be applied. To our knowledge, the mechanical properties of LS's attachment have not been explored.

### Krill swimming

Krill swim, which could affect filtering in several ways. Because krill are negatively buoyant, they typically swim continuously, and swimming while in the pouch would likely prevent them from settling to the bottom of the pouch away from the baleen window (located just under the upper jaw). In this respect, swimming augments clogging. By contrast, the combined effects of thousands of swimming krill could possibly be sufficient to mix the water in the pouch sufficiently to keep krill from clogging the baleen mat. In particular, clogging could be avoided if krill actively swim away from the baleen window. It seems unlikely, however, that krill can reliably muster the speed of 0.67 m s^−1^ required to avoid being trapped on the baleen filter. In a flow chamber, Antarctic krill (*E. superba*) could not maintain a steady swimming speed above 0.17 m s^−1^ (Swadling et al., 2005). They can reach a maximum speed of 0.30–1.00 m s^−1^ during an escape, but the average speed during the escape is only 0.28 m s^−1^, again insufficient to offset the speed at which water is expelled (Kils, 1982; Connor and Webster, 2023). It is also possible that live krill interact differently with the multi-layered, flexible mat of baleen fringes than the dead krill did with the planar, inflexible grid in our experiments, perhaps leading to lower hydraulic resistance. Again, only further research can address these possibilities.

### Conclusions

Our measurements of the hydraulic resistance imposed by krill as they accumulate on baleen suggest that clogging of the baleen window may impose a challenge for rorqual whales as a result of their swarm-engulfment strategy. If filtration occurs in the simplest manner (i.e. a dead-end filter), with krill uniformly spread on the baleen window, the pressure required to expel water in the interval observed in nature exceeds what can be reasonably produced by the muscles and blubber of the buccal pouch. Gradual accumulation of prey and escape swimming by krill are unlikely to effectively reduce the required pressure. By contrast, the effects of parallel resistance could alleviate the problem, but only if a substantial fraction of the window's area has a low hydraulic resistance. In turn, that requires that that fraction be maintained nearly free of krill, and it is unclear how that might be accomplished.

Our results highlight yet another way in which our understanding of rorqual whales is incomplete. It has been proposed that the evolution of whales' immense body size was facilitated by their ability to engulf entire, dense aggregations of prey. But even then, they need to make multiple gulps per dive to fuel their metabolism, limiting the time available to filter each gulp. Our calculations suggest that simply sieving prey would stretch that interval beyond what is viable, leaving a gap in our mechanistic explanation of body size. To the long list of important research areas regarding these magnificent creatures should be added: (1) a better understanding of the physiology of buccal-pouch musculature and (2) detailed measurement of the dynamics of water motion within the pouch during feeding.

## Figures and Tables

**Fig. A1.**
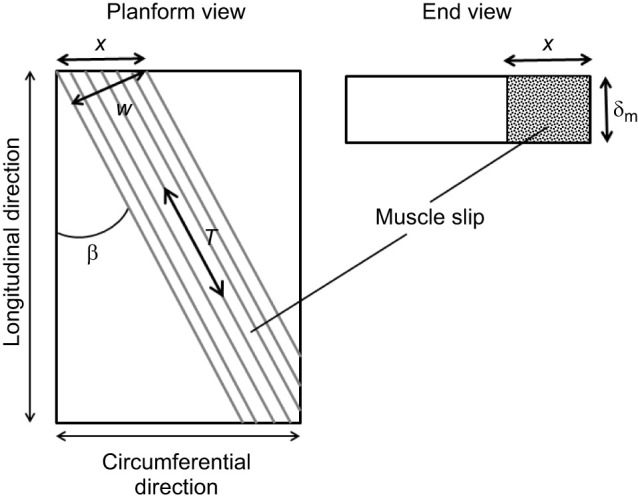
**Geometry used to calculate the circumferential force imposed by muscular contraction.** A schematic slice of the buccal pouch showing a single slip of oblique muscle. In the real pouch, for each muscle oriented with its axis at angle β to the whale's longitudinal axis, there is a slip oriented with its axis at −β. Both planform and end-on views of the slice are shown. δ_m_ is the thickness of the muscle layer; *x* is the length of a slice though the muscle in the circumferential direction; *w* is muscle width; and *T* is the tension developed by the contracting muscle.

**Fig. A2.**
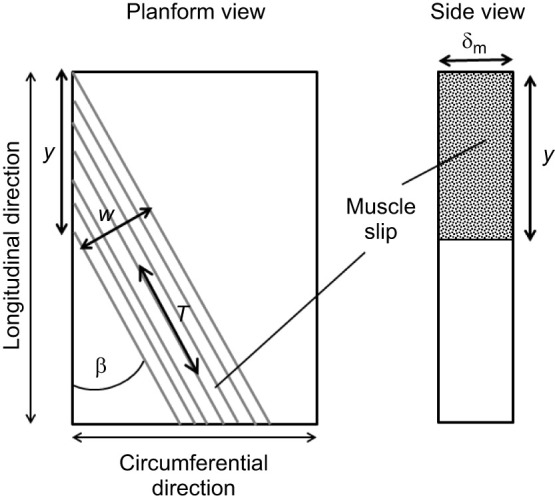
**Geometry used to calculate the longitudinal force imposed by muscular contraction.** A schematic slice of the buccal pouch showing a single slip of oblique muscle. In the real pouch, for each muscle oriented with its axis at angle β to the whale's longitudinal axis, there is a slip oriented with its axis at −β. Both planform and side-on views of the slice are shown. δ_m_ is the thickness of the muscle layer; *y* is the length of a cross-section through the muscle in the longitudinal direction; *w* is muscle width; and *T* is the tension developed by the contracting muscle.

## APPENDIX 1

### Forces applied by muscles

Consider a rectangular slice of the oblique muscle stratum with one side parallel to the buccal cavity's circumference and the other parallel to the cavity's longitudinal axis (Fig. A1). Within that slice, we consider a single slip of muscle of width *w* and thickness δ_m_, with its axis at an angle β to the slice's longitudinal direction. (In the actual oblique substratum, each muscle slip oriented at angle β is matched by another oriented at angle −β, but the effect is the same.) The tension developed by the contracting muscle, *T*, is the product of muscle stress, σ_m_ (Pa), and the slip's cross-sectional area (δ_m_*w*, m^2^):

(rmA1)

From the geometry of the situation, we see that *x*, the width of the muscle slip measured in the circumferential direction, is *w*/cosβ. Thus, *w*=*x*cosβ and:

(rmA2)

The component of *T* directed in the longitudinal direction is *T*cosβ, so *F*_L,m_, force acting in the longitudinal direction, is:

(rmA3)

This relationship can be generalized to the entire oblique stratum, in which case *x* is circumference *C*_1_ from Fig. 1A:

(rmA4)

The same logic can be applied to the slice of OS shown in Fig. A2. In this case, *w*=*y*sinβ, and tension in the muscle is:

(rmA5)

The component of *T* directed in the circumferential direction is *T*sinβ, so *F*_C_, force acting in the circumferential direction, is:

(rmA6)

This, too, can be generalized to the entire OS:

(rmA7)

where *C*_2_ is the circumference shown in Fig. 1B.

## APPENDIX 2

### Orientation of oblique muscle fibers

The pressure applied to water in the buccal pouch by the combined force of the longitudinal component of OS muscle contraction and the longitudinal contraction of blubber is:

(rmA8)

where σ_L,b_ is longitudinal stress in the blubber, δ_b_ is stretched blubber thickness, and *k*_1_=(δ_m_*C*_1_)/*A*_1_ (dimensionless) and *k*_2_=(σ_L,b_δ_b_*C*_1_)/*A*_1_ (Pa).

Pressure applied by the combined action of stretched blubber and the circumferential component of muscle contraction is:

(rmA9)

where σ_C,b_ is circumferential stress in the blubber, and *k*_3_=(δ_m_*C*_2_)/*A*_2_ (dimensionless) and *k*_4_=(σ_C,b_δ_b_*C*_2_)/*A*_2_ (Pa).

Because both *P*_C_ and *P*_L_ are applied to the same water, they must be the same. Setting them equal, we can solve for cosβ:

(rmA10)

Recalling that (sinβ)^2^+(cosβ)^2^=1:

(rmA11)

Solving for (cosβ)^2^:

(rmA12)

(rmA13)

For an average fin whale, δ_m_=0.046 m, *C*_1_=9.16 m and area *A*_1_ is 6.81 m^2^; *C*_2_=15.5 m, δ_b_=0.046 m and area *A*_2_ is 35.2 m^2^ (see Fig. 1). σ_C,b_=53.6 kPa and σ_L,b_=15.9 kPa. For average contraction stress (200 kPa), β=58.7 deg. For maximal muscle stress (1000 kPa), β=59.8 deg. β is approximately 45 deg when the blubber is relaxed (Orton and Brodie, 1987). The higher β implied by our calculations is consistent with the predominance of circumferential extension to which the blubber is subjected during a gulp (circumferential λ=2.58 versus λ=1.38 for longitudinal extension) (Shadwick et al., 2013).

## APPENDIX 3

### Coronal plane

There are three planes from which the interaction between pouch morphology and buccal pressure can be productively examined. Our analysis takes advantage of the simplicity offered by the sagittal and transverse planes. The remaining option – a coronal plane perpendicular to the other two and running through the plane formed by the lower jaw – is theoretically useful, but in practice would require morphological details that are currently unavailable.

While our model of the buccal pouch is sufficient for accurate estimation of volume, area and circumference, it does not accurately portray morphology in the coronal plane, and that is problematic. To see why, we first revisit the balance between buccal pressure *P*, cross-sectional area *A*, and the forces applied by blubber and muscles *F* (Eqns 3 and 6):

(rmA14)

where *F*=σδ*C* (muscle stress×muscle thickness×circumference). Implicit in that force balance is the assumption that the force applied by blubber and muscle acts perpendicular to the plane in which area is measured. Because the transverse and sagittal planes of our analysis are taken through the point of maximum pouch extension, that assumption is inherently true.

By contrast, if blubber and muscles do not contact the coronal plane at right angles, the pressure applied by each small bit of circumference is proportional to *F*sinθ where θ is the angle between the force vector and the coronal plane. In fin and blue whales, that angle varies around the circumference and from visual estimates is typically substantially different from 90 deg. If, for instance, θ is 60 deg on average, the pressure resulting from blubber and muscle would be 13% less than it would be for perpendicular attachment; if θ is 45 deg, it would be 29% less. Thus, without accurate knowledge of θ for the entire coronal circumference, it is difficult to accurately estimate pressure from the perspective of the coronal plane.

That difficulty is compounded by the fact that, in the oblique stratum (OS), muscle fibers lie in a cross-striated pattern, such that the force applied is direction dependent. For example, if fibers were oriented at 90 deg to each other (each 45 deg to the long axis), when one set of fibers (half the total) is oriented parallel to the direction of pull, the other half could offer no tension in that direction. The effective force applied by OS muscles thus depends on the angle at which fibers are oriented relative to the plane of the area on which they act. That fiber angle is taken into account in our analyses for the transverse and sagittal sections (Appendix 1 and Appendix 2). By contrast, the angle at which fibers connect to the body in the coronal plane is difficult to estimate from the morphological data available. If our estimate of fiber angle (59 deg from the pouch's long axis) is accurate and applies to muscles at the pouch's periphery, the force applied by the OS could range between 27% and 73% of those based on σ and thickness alone. Without additional information regarding the insertion of OS muscles (which is not currently available), how that percentage would average out around the circumference of the coronal section is unclear. In short, it would be difficult to accurately estimate *F* in Eqn A14.

Lastly, in the coronal section, only the anterior third of the pouch is attached to the rigid jaw; the posterior two-thirds attach to the body wall. As with any pressure potentially applied by the longitudinal stratum (LS) muscles (discussed in the main text), the force applied perpendicular to the coronal plane by this posterior portion of the pouch will depend on the compliance of the body. Any compliance would again reduce the estimated pressure, but accurately estimating the extent of the reduction would be problematic.

Taken together, these issues currently make it difficult to accurately estimate pouch pressure from the coronal perspective. It will be valuable to revisit this alternative approach when additional morphological detail is available.

## APPENDIX 4

### Pressure as a function of muscle contraction stress

From Eqn A8 we see that:

(rmA15)

Substituting Eqn A12 for (cosβ)^2^, we can solve for σ_m_. This gives:

(rmA16)

Using the typical values from Appendix 2 and expressing both pressure and muscle stress in pascals, we find that σ_m_ needed to exert a given *P* is:

(rmA17)

## APPENDIX 5

### Gradual accumulation of krill

By analogy to Ohm's law for the flow of electricity, we can use our experimental measurements to calculate the resistivity, *R* (Pa s m^−1^), of krill to flow:

(rmA18)

Here d*V*/d*t* is the volumetric rate at which water drains from the apparatus shown in Fig. 2, and *A* is the cross-sectional area of the tube; *u* is the speed of outflow. Measured resistivities are shown in Fig. A3A. Linear fits to these data allow us to estimate the resistivity for each thickness of krill at the pressures applied by average and maximal contraction of the buccal pouch (4.1 and 17.7 kPa, respectively) (Fig. A3B). In addition, extrapolations from our measurements of flow as a function of pressure (Fig. 3B) allow us to estimate the pressures required to achieve an outward current of 0.67 m s^−1^ for the three thicknesses of krill used in our experiments. From those pressures and that current, we can estimate resistivity at high pressure (Fig. A3B). These estimated resistivities allow us to model the effects of gradual krill accumulation.

The average gulp of a fin whale (60 m^3^) contains 90 kg of krill, so ρ_k_, the mass of krill in a cubic meter of engulfed water, is 1.5 kg m^−3^. The packing density of krill, ρ_p_, the mass of krill per cubic meter of packed krill, is 784 kg m^−3^. The volume of krill per m^3^ of water inside the buccal pouch is ρ_k_/ρ_p_. *A*_bal_ is the area of the baleen window. Thus, δ_k_=ρ_k_/(ρ_p_*A*_bal_) is the uniform thickness of krill accumulated per volume of water expelled. The volume of water expelled in a short interval of time, Δ*t*, is *u*(*t*)*A*_bal_Δ*t*, where *u*(*t*) is the speed of water flowing outward at time *t.* The increment in δ_k_ in Δ*t* is thus:

(rmA19)

From the definition of resistivity (Eqn A18):

(rmA20)

where *P* is the pressure imposed by blubber and muscle, and *R*(δ_k_) is the resistivity due to both the baleen itself and the accumulated krill. Thus:

(rmA21)

The thickness of krill can be integrated through time in a series of *n* steps where each step encompasses an interval of Δ*t* seconds:

(rmA22)

The total volume of a gulp is expelled in 31 s. Consequently, setting δ_k_(0)=0 and using a time step of Δ*t*=0.1 s, we carry out this calculation for *n*=1 to 310. For each δ_k_, there is a corresponding *u* from Eqn A20. We can estimate *R*(δ_k_) using our empirical measurements (Fig. A3B). For *P*=4.1 kPa, *R*(δ_k_)=3485×δ_k_+89.61 [where *R*(δ_k_) is measured in kPa m s^−1^, δ_k_ in m; *r*^2^=0.868]. For *P*=17.7 kPa, *R*(δ_k_)=2224×δ_k_+40.46 (*r*^2^>0.999). For *P*=26.0 kPa, *R*(δ_k_)=208×δ*_k_*+34.77 (*r*^2^=0.918). These results are shown in Fig. 4.

## APPENDIX 6

### Pressure during pouch contraction

To estimate the effect of contraction on the pressure in a whale's buccal pouch, we take as a useful model a thin-walled circular cylinder with an elastic wall (blubber) of fixed volume, *V*_b_, and a fixed length, *L* (fixed because the VGB is very stiff longitudinally). The cylinder has radius *r*, which at rest is *r*_0_. The volume of blubber at any radius is equal to its transverse cross-sectional area (circumference×thickness, δ) times its length:

(rmA23)

So, thickness is:

(rmA24)

As water is pumped into the cylinder (we assume its ends are covered), its radius increases, stretching the blubber. The extension ratio, λ, of the stretched blubber is its stretched circumference divided by its resting circumference:

(rmA25)

If the blubber is elastic, the stress (σ_b_, force per area) in the stretched tissue increases with increasing λ. We can model that relationship as:

(rmA26)

where λ−1 is strain (change in length per resting length) and *E* is a constant of proportionality. (When α=1, the material is linearly elastic, and *E* is the elastic modulus.)

The circumferential force exerted by the stretched blubber on the enclosed water is equal to the stress in the blubber times its longitudinal cross-sectional area (2*L*δ). The pressure, *P*, imposed on the enclosed fluid is that force divided by the longitudinal cross-sectional area of the water in the cylinder (2*rL*):

(rmA27)

Substituting Eqns A24 (for thickness) and A26 (for stress) into Eqn A27 yields pressure as a function of radius:

(rmA28)

For fin whale VGB stretched circumferentially to strains <2, α is approximately 2.4 (see fig. 5 of Orton and Brodie, 1987). Setting α=2.4, pressure (normalized to the pressure at maximum extension ratio in the buccal pouch) is shown as a function of strain in Fig. 7.
